# CTLA-4 (+49A/G) Polymorphism and Type-1 Diabetes in Turkish Children

**DOI:** 10.4274/Jcrpe.879

**Published:** 2013-03-21

**Authors:** Fatih Çelmeli, Doğa Türkkahraman, Deniz Özel, Sema Akçurin, Olcay Yeğin

**Affiliations:** 1 Antalya Education and Research Hospital, Department of Pediatric Immunology, Antalya, Turkey; 2 Antalya Education and Research Hospital, Department of Pediatric Endocrinology, Antalya, Turkey; 3 Akdeniz University Faculty of Medicine, Department of Medical Statistics, Antalya, Turkey; 4 Akdeniz University Hospital, Department of Pediatric Endocrinology, Antalya, Turkey; 5 Akdeniz University Hospital, Department of Pediatric Immunology, Antalya, Turkey

**Keywords:** : CTLA-4, polymorphism, type-1 diabetes

## Abstract

**Objective**: To evaluate the contribution of cytotoxic T-Iymphocyte antigen-4 (CTLA-4)+49A/G polymorphism to the susceptibility to type-1 diabetes (T1D) in Turkish children.

**Methods:** A case-control study was designed to include 91 Turkish children with T1D and 99 healthy controls. CTLA-4 (+99A/G) gene polymorphism typing was done by PCR amplification followed by restriction fragment length polymorphism method.

**Results:** The genotype and allele frequencies of the CTLA-4 (+99A/G) polymorphism in patients with T1D were not different from those in the controls (p>0.05). The allele frequency of G was 36.2% in patients with T1D, and 31.8% in controls (p>0.05). Additionally, this polymorphism was not associated with the clinical and laboratory characteristics of the patients with T1D (p>0.05).

**Conclusions:** Our case-control study suggests that the CTLA-4 (+99A/G) gene polymorphism is not associated with T1D in the Turkish population.

**Conflict of interest:**None declared.

## INTRODUCTION

Diabetes mellitus is a group of metabolic diseases characterized by chronic hyperglycemia resulting from defects in insulin secretion, insulin action, or both. Type-1 diabetes (T1D) is an insulin-dependent form of diabetes with high mortality and morbidity rates, which usually begins in childhood and adolescence. Most cases are primarily due to T-cell-mediated pancreatic islet β-cell destruction, and the patient becomes clinically symptomatic when approximately 90% of pancreatic beta-cells are destroyed (1). Serological markers of the autoimmune process including islet cell, glutamic acid decarboxylase (GAD), IA-2, or insulin autoantibodies (IAA) are present in 85-90% of individuals when fasting hyperglycemia is detected (2).

A variety of genetic predisposing factors and contributing factors are known to influence the pathogenesis of T1D. There is some evidence suggesting that the susceptible genes to T1D are associated with amplification of the immune response and rate of progression of the disease. The role of these genes appears to be more important during childhood than during adult life (3). In several studies, it has been shown that more than 40 genetic loci are associated with T1D (4). Many of the susceptibility genes are located within the HLA locus on chromosome 6p21, known as IDDM1 (5). Another significant susceptibility locus (IDDM12) maps to cytotoxic T-lymphocyte antigen-4 (CTLA-4) gene region of chromosome 2q33 (6). IDDM12 has also been implicated in systemic lupus erythematosus, autoimmune thyroiditis, celiac disease, and rheumatoid arthritis (7). IDDM12 contains a cluster of ?T-lymphocyte-regulating genes including CD28, CTLA-4, and inducible co-stimulator (ICOS). CTLA-4 is a member of ?the immunoglobulin superfamily that is expressed on the surface of activated T-cells and downregulates T-cell function, whereas CD28 enhances T-cell proliferation. Binding of ?CTLA-4 to the B7 receptor limits the proliferation of T-cells and terminates the ongoing immune response (8). In many molecular epidemiologic studies, CTLA-4 (+49A/G) single nucleotide polymorphism (SNP) that causes a threonine-to-alanine substitution in codon 17 has been found to be associated with genetic susceptibility to T1D in several populations, although conflicting data also exist in populations of different ethnic backgrounds (9). The aim of this study was to evaluate the contribution of CTLA-4 (+49A/G) polymorphism to the susceptibility to T1D in Turkish children. 

## METHODS

Ninety-one unrelated Turkish patients aged between 3 and 19 years with T1D diagnosed in our outpatient clinic were included in the study. The diagnosis of T1D was based on the blood glucose level as per the World Health Organization diagnostic guidelines. Clinical symptoms, absolute insulin-dependency, presence of diabetes-related autoantibodies (DRA), islet cell antibodies (ICA), GAD antibodies (GADA), and IAA were also considered in the diagnosis. Demographic characteristics, clinical presentations, presence of other autoimmune diseases, HbA1c levels, and diabetic autoantibody positivity at presentation were recorded in all patients.

The control group consisted of 99 unrelated healthy subjects without T1D and known autoimmune disease, living in the same geographical area, and having the same ethnic origin as the patients. The Local Medical Ethics Committee approved the study, and informed consent was obtained from the parent or guardian of each participating subject.

Genomic DNA was extracted from the peripheral blood of individuals using an MBI Fermentas DNA isolation kit according to the instructions of the manufacturer. Genotyping of polymorphic restriction sites in the CTLA-4 gene exon 1 position 49 which encodes a threonine (GCC) to alanine (ACC) substitution at codon 17 was done by using PCR amplification followed by the restriction fragment length polymorphism (RFLP) method. Amplification of a 162 bp genomic region of CTLA-4 gene was performed by using forward (5’-GCTCTACCTCTTGAAGACCT-3’) and reverse (5’-AGTCTCACTCACCTTTGCAG-3’) primers described previously. A 0.25 μg genomic DNA was amplified in each 25 μl PCR reaction containing 50 μM of each dNTPs (Boehringer, Germany), 2 U of Taq DNA polymerase, 2.5 μl of 10?PCR buffer, 0.8 μM of each primer. The reaction mixture was first heated at 94 0C for 4 min and amplification was done for 33 cycles in a PCR thermocycler by denaturation at 94 0C for 45 s, annealing at 60 0C for 45 s and extension at 72 0C for 45 s at each cycle.

RFLP analysis was done using FastDigest BbvI (Fermentas, Germany) in 30 μL total volume by mixing: 10 μL of PCR products, 1.0 μL of BbvI restriction enzyme, 2.0 μL 10 X FastDigest green buffer, and 17 μL nuclease-free water. The mixture was incubated at 37 0C for 10 min followed by heating at 65 0C for 10 min. DNA fragments were resolved in 2.0% agarose gels. The A allele does not create restriction site (162 bp), while the G allele creates restriction site producing two fragments (88 bp and 74 bp).

**Statistical Analysis**

The statistical analyses were performed using SPSS 10.0 software package. Mann-Whitney U, chi-square, Kruskal-Wallis and student’s t tests as well as one-way ANOVA were used to compare the differences. Quantitative data were presented as mean±SD. A p-value of less than 0.05 was considered significant.

## RESULTS

The study was conducted on 91 patients with T1D (39 male, 52 female) aged between 3 and 19 years. Mean age at diagnosis was 8.5 years (range; 1.5-18 years). Mean follow-up period was 3.2 years (range; 0.2-18 years). The control group consisted of 99 unrelated healthy subjects (47 male, 52 female) aged between 9 and 30 years. There was no statistically significant difference between the control and patient groups with respect to gender or age (p>0.05). Of the patients with T1D, 43 (47.3%) presented with diabetic ketoacidosis (DKA) and 48 (52.7%) with hyperglycemia (with or without ketosis). T1D was accompanied by an autoimmune disease (Hashimoto’s thyroiditis, celiac disease, and vitiligo) in 19 (20.9%) of the patients. DRA positivity (in at least one of the three autoantibodies) was present in 66.1% (39/59) of the patients in whom DRAs measurements were performed. Clinical and laboratory characteristics of the patients at diagnosis are presented in Table 1.

As shown in Table 2, the distribution of CTLA-4 genotype and allele frequencies did not differ significantly between patients and controls (p>0.05). No single genotype or allele was associated with an altered risk for T1D. Forty (43.9%) of the patients were heterozygous for A/G, 38 (41.8%) were homozygous for A and 13 (14.3%) were homozygous for G, whereas the corresponding numbers and frequencies of A/G, A/A, and G/G genotypes in healthy controls were 49 (49.5%), 43 (43.4%), and 7 (7.1%), respectively. In patients, the allele frequency of G was comparable to that in the controls (36.2% vs. 31.8%, p >0.05).

Clinical features and laboratory findings of the patients at diagnosis in relation to CTLA-4 (+49A/G) genotypes and alleles are presented in Table 3. There was no statistically significant difference between the variables (age, gender, presence of DKA, HbA1c level at diagnosis, presence of other associated autoimmune diseases, and DRA positivity) and the CTLA-4 (+49A/G) genotypes and allele frequencies (p>0.05).

## DISCUSSION

We investigated the +49A/G polymorphism because it has been the most widely analyzed CTLA-4 variant in T1D patients from several ethnic populations. In addition, it is the only known SNP that causes an amino acid change (threonine to alanine) and one that is associated with altered protein expression and T-cell activation. Our results do not support the involvement of CTLA-4 gene in the pathogenesis of T1D in the Turkish population. This contrasts with the positive associations that have been reported for the +49A/G polymorphism in populations including Spanish, French, Korean, Italian, Mexican-American (10), Belgian (11), Japanese (12), Estonian (13), Iranian (14), and Egyptian (15). On the other hand, no association has been reported in many populations including Chilean (16), Chinese and British (10), Japanese (17), Portuguese (18), Brazilian (19), and Azerbaijani (20). These contradictory results can be explained by the genetic heterogeneity among the studied populations, by the different environmental factors involved in the pathogenesis of T1D, by the limitations of the studies or other methodological issues. Moreover, the CTLA-4 (+49A/G) SNP may not be the real disease-associated variant but rather a marker in linkage disequilibrium with the causal variant, and these inconsistent findings may illustrate the variable strengths of linkage disequilibrium in different populations (18).

The distribution of the CTLA-4 exon 1 polymorphism among Asians and Caucasians shows a clear difference. According to a large meta-analysis (9), the pooled frequency of the G allele was 43.3% among control subjects (by race, frequencies were 55.4%, 36.2%, 33.6%, 20.6%, and 45.2% among controls of Asian, European, North African/Middle Eastern, Sub-Saharan African, and Pacific Asian descent, respectively). The overall pooled prevalence of G/G homozygosity was 20.4% (33.4%, 12.8%, 8.9%, 5.7%, and 22.3% in the five racial descent groups, respectively). The overall pooled prevalence of G/A heterozygosity was 44.8% (44.1%, 46.8%, 49.4%, 31.2%, and 45.7% in the five racial descent groups, respectively). Additionally, analysis of genotypes suggested that G/G homozygous individuals are at a 2-fold higher risk of developing T1D. Our results are concordant with the findings of this meta-analysis in a way that genotype and allele frequencies of the Turkish population investigated in this study are in between those reported for European and Middle East populations.

In some studies, it has been reported that CTLA-4 polymorphisms are associated with the clinical characteristics of patients with T1D. Abe at al (21) reported that +49A/G polymorphism is associated with ICA512 antibody positivity and with presence of DKA at diagnosis. Balic et al (22) showed that +49A/G polymorphism could confer a genetic risk for T1D, particularly with G allele dosage in younger individuals. Their data also suggested that the association of CTLA-4 with T1D is more striking in patients carrying the G allele of +49A/G polymorphism, with higher episodes of ketoacidosis and higher glycemic levels at diagnosis. However, the results of our study showed no association between CTLA-4 (+49A/G) polymorphisms and the clinical characteristics of patients with T1D. Similar results have also been reported by others (15,18).

In conclusion, CTLA-4 (+49A/G) gene polymorphism was not associated with T1D in the Turkish population studied. However, more studies with a larger study population are needed to confirm these findings.

## Figures and Tables

**Table 1:**
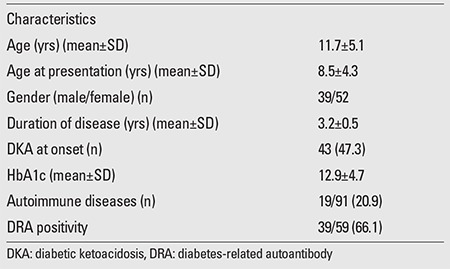
Clinical and laboratory characteristics of the patients at diagnosis

**Table 2:**
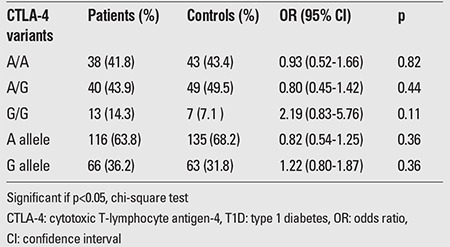
Genotype and allele frequencies of CTLA-4 (+49A/G) polymorphism in patients with T1D versus controls

**Table 3:**
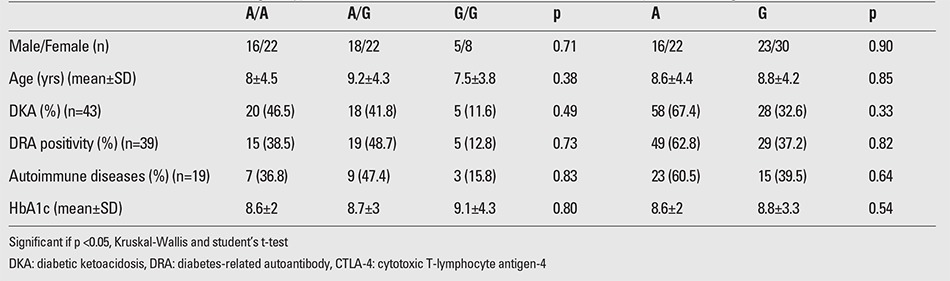
Distribution of CTLA-4 (+49A/G) genotypes and alleles in relation to the clinical features of the patients at diagnosis
